# Resolving charge and glycosylation variants of cetuximab combining offline ion-exchange and hydrophilic interaction chromatography–HRMS

**DOI:** 10.1007/s00216-026-06519-w

**Published:** 2026-04-27

**Authors:** Annika A. M. van der Zon, Andrea F. G. Gargano

**Affiliations:** 1 Analytical Chemistry Group, Van ’t Hoff Institute for Molecular Sciences, University of Amsterdam, Science Park 904, 1098 XH Amsterdam, The Netherlands; 2 Center of Analytical Sciences Amsterdam, Science Park 904, 1098 XH Amsterdam, The Netherlands

**Keywords:** Monoclonal antibodies, 2D-LC, Charge heterogeneity, Glycosylation, HRMS

## Abstract

Biopharmaceuticals, such as monoclonal antibodies (mAb), are complex molecules that require thorough analysis due to post-translational modifications, including charge variants and glycoform patterns. When analyzing intact mAbs, single liquid chromatography (LC) mass spectrometry (MS) methods often lack the resolution to distinguish this heterogeneity because they offer limited separation in both chromatographic and mass dimensions. Consequently, these methods struggle to detect low-abundance species, highlighting the importance of 2DLC-MS workflows. Here, we demonstrate the potential of the offline ion exchange chromatography (IEC)–hydrophilic interaction chromatography (HILIC)–MS method to effectively separate charge and glycoform variants of complex mAbs. Cetuximab served as the model antibody due to its multiple charge variants (arising from sialylation and C-terminal lysine clipping) and four glycosylation sites (in both Fab and Fc regions). Charge variants were first separated using non-volatile IEC and collected as individual fractions. These fractions were then analyzed using a low-flow HILIC-MS setup to increase sensitivity for low-abundance species. A RPLC trap column was used to desalt and reduce the volume transferred to the HILIC separation. The IEC-HILIC results showed that both Fc- and Fab-associated glycoforms of cetuximab were resolved to a level not achievable with HILIC-MS alone, allowing deeper insights into charge heterogeneity and glycosylation. In addition to resolving combined Fab/Fc glycoforms, the method also distinguished Fc-only glycoforms from the main isoform. This orthogonal IEC-HILIC method provides additional insights into charge and glycosylation heterogeneity, enabling a more comprehensive analysis of cetuximab. It provides an effective approach for detailed characterization of charge variations and glycoform diversity in next-generation protein therapeutics.

**Graphical abstract:**

Orthogonal separation of IEC (charge variants) with offline HILIC-MS (glycosylation and characterization of proteoforms)

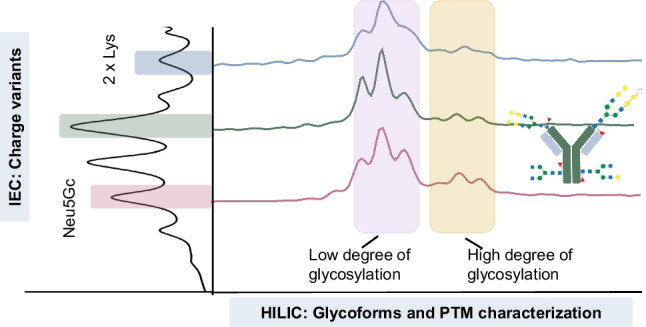

**Supplementary Information:**

The online version contains supplementary material available at 10.1007/s00216-026-06519-w.

## Introduction

The landscape of biopharmaceutical development is rapidly evolving from standard monoclonal antibodies (mAbs) toward increasingly complex modalities, including Fc-fusion proteins, and antibody-drug conjugates [1, 2]. As these therapeutics become more complex, they exhibit a combinatorial diversity of post-translational modifications (PTMs) that can critically impact safety, efficacy, and pharmacokinetics [3]. Among these, charge variants (arising from deamidation, sialylation, or C-terminal lysine clipping) and glycan heterogeneity are critical quality attributes (CQAs) that must be rigorously monitored. In particular, the glycoform heterogeneity of biopharmaceuticals may cover a vast chemical space [4], with cases in which 10^6^ glycoforms of a biopharmaceutical have been described [5]. In next-generation therapeutics, these attributes are often superimposed; for instance, a specific acidic charge variant may possess a distinct, immunogenic glycan profile.

Currently, the characterization of charge and glycan heterogeneity relies on separate, non-orthogonal one-dimensional liquid chromatography (LC) methods in which mass spectrometry (MS) plays an important role in the mass detection. As a result, LC-MS techniques that monitor each of these chemical distributions separately may be inadequate for thoroughly characterizing the structure of complex sample therapeutics as they may lose the correlation between chemical distribution (e.g., charge and glycosylation variants). Ion-exchange chromatography (IEC) is the gold standard for separating charge variants [6, 7]. However, it offers no selectivity for neutral glycoforms and is inherently difficult to couple directly with MS due to the high concentrations of non-volatile salts (> 200 mM) typically required [8]. Recent progress has enabled coupling IEC with MS using pH gradients and volatile buffers [9]. Using IEC-MS, Füssl et al. characterized the heterogeneity of cetuximab, a complex mAb with four glycosylation sites (two in the Fab region and two in the Fc region) [10, 11]. By aligning intact-level IEC-MS and CE-MS data with released glycan profiles, they successfully matched glycoform combinations to the expected migration times of various charge variants, assigning more than 100 isoforms of cetuximab.

Reversed-phase liquid chromatography (RPLC) is often considered the reference method for intact protein and mAb analysis. While RPLC serves as an effective MS desalting interface and a fast second LC dimension [12, 13], it lacks the selectivity to resolve glycoforms or charge variants. In contrast, hydrophilic interaction chromatography (HILIC) has emerged as a powerful technique for resolving glycoforms of glycoproteins. Until recently, HILIC was largely limited to relatively small glycoproteins (< 50 kDa) [14–22]. However, we recently demonstrated intact glycoform separation of mAbs using HILIC-MS with a new material, acrylamide monoliths [23]. This intact-level approach preserves the combinatorial context of the glycoforms, information that is lost during bottom-up or middle-up digestion protocols. However, the direct application of HILIC-MS to highly complex therapeutics is hindered by the co-elution of charge variants, which can obscure low-abundance species and severely complicate spectral deconvolution.

To address this, a multidimensional approach coupling the charge-based selectivity of IEC with the glycan selectivity of HILIC would, in principle, be highly effective. Such an orthogonal setup would allow for the specific assignment of glycan profiles to distinct charge variants, resolving proteoforms that co-elute in single-dimension separations. However, the online coupling of IEC and HILIC for intact proteins remains a significant analytical challenge due to severe solvent incompatibility [24]. IEC requires highly aqueous, salt-rich mobile phases [25], whereas HILIC separations rely on high-organic starting conditions (e.g., 60–80% acetonitrile) [26]. Therefore, the direct transfer of IEC fractions to a HILIC column typically results in analyte breakthrough or peak distortion, preventing effective trapping and separation. As a result, despite the high orthogonality of the two methods, the successful implementation of two-dimensional LC combining IEC and HILIC has been demonstrated in only a limited number of cases [27–30].

In this work, we present an orthogonal IEC–HILIC–MS workflow that overcomes this solvent incompatibility, enabling an extended analysis of the heterogeneity of complex mAb biopharmaceuticals. To realize the offline coupling of IEC with HILIC, we utilized an RPLC trap column interface to capture, desalt, and transfer IEC fractions into a low-flow HILIC-MS dimension [31], ensuring compatibility between the high-salt first dimension and the high-organic second dimension. We applied this methodology to cetuximab, a highly complex chimeric IgG_1_ bearing four N-glycosylation sites and significant charge heterogeneity [32]. While previous studies have inferred glycoform distributions in cetuximab based on peptide mapping or separate migration times [30, 33, 34]. This approach enables the direct, intact-level observation of specific glycoform-charge variant pairings. This workflow provides a blueprint for high-resolution separations that may be needed to characterize next-generation protein therapeutics, where charge and glycosylation profiles are linked.

## Materials and methods

### Chemicals

ACN and trifluoroacetic acid (TFA) were purchased from Biosolve (Valkenswaard, The Netherlands). Ultrapure water was obtained from a MilliQ purification system (Merck Millipore, Burlington, USA) or an Arium 611UV system (Sartorius, Göttingen, Germany). Sodium hydroxide, sodium chloride, and 2-(N-Morpholino)ethane sulfonic acid (MES) were purchased from Sigma-Aldrich (Zwijndrecht, The Netherlands). Cetuximab (5 mg·mL^−1^) was obtained from Amsterdam University Medical Center. Cetuximab was diluted to 1 mg·mL^−1^ in water prior to the IEC measurement.

### Instrumentation

#### IEC-UV

An Agilent 1290 Infinity II Bio LC system (Agilent Technologies, Waldbronn, Germany) was used (Fig. S1). For the IEC separation (method conditions adapted from [25]), Waters BioResolve strong cation exchange (SCX) mAb column (3 µm, 2.1 × 100 mm) was used with the elution buffers consisting of 20 mM MES in 95:5 water: ACN (pH 5.7) (mobile phase A) and 20 mM MES + 125 mM NaCl in 95:5 water: ACN (pH 5.7) (mobile phase B). The pH was adjusted using 1 M sodium hydroxide and controlled by using FiveEasy Plus pH meter (Mettler Toledo, Tiel, The Netherlands). All measurements were conducted at room temperature (25 °C) with a flow rate of 100 µL·min^−1^. The gradient started at 10% B and increased linearly to 23% over 2 min. Then, it increased further to 36% B over 30 min, maintaining this level for a further 2 min. The column was then cleaned at 100% B for 5 min, after which there was a 10-min re-equilibration time at 0% B. The injection volume was 5 µL, and the detection wavelength was set to 280 nm.

#### Fraction collection

First, the cetuximab was run without fractionation to set the fractionation time windows. Then, five fractions were taken based on time windows (Table S1). The threshold was set to 200 mAU, with up and down slopes of 5.00 mAU. The fraction volumes were around 102 and 138 µL. After fractionation, the fractions were immediately stored at −80 °C.

#### HILIC-MS

For the HILIC-MS, the complete setup and methodology were used as reported here [31] (Fig. S2). However, for cetuximab, the gradient was optimized. It started at 95% mobile phase B (0.1% (v/v) TFA in 2:98 water:ACN) and 5% mobile phase A (0.1% (v/v) TFA in 98:2 water:ACN) and ramped down to 72% B in 1 min. Then, it gradually decreased to 67% B over 20 min, followed by a decrease to 50% B in 5 min and further down to 20% B in 1 min. To clean the column, two washing cycles were implemented, starting from 80 to 20% B for each 1 min, followed by re-equilibration at 95% B for 9.9 min (a factor of 2). For charge variant peaks, 20 µL of the sample was injected, whereas for the main isoform, only 2 µL was used.

For the direct injection of cetuximab, 200 ng (100 µg·mL^−1^, 2 µL) was injected. The trapping time was set to 10 min (40 µL). To ensure there was no carryover present, three blanks were run between the mAb injections. The same MS settings were used as described here [31].

### Data processing

The Agilent instrumentation was managed by OpenLab ChemStation (Agilent Technologies, Waldbronn, Germany). The QExactive Plus was controlled by Xcalibur (Thermo Fisher Scientific, Bremen, Germany). The raw MS data were visualized in Freestyle. For the deconvolution of the raw MS data (3000–6000 mass-to-charge (*m/z*)), the latest version of Unidec (15-01-2026) (University of Arizona, Phoenix, USA) was used with the following parameters: a sample mass rate of 0.1 Da, a picking threshold of 0.05 Da, and a range of 0.1 Da [35]. The retention times of the glycoforms were found by the Apex. Raw MS data can be found here: 10.5281/zenodo.18401232.

## Results and discussion

Cetuximab (IgG_1_) was selected as the model therapeutic for this study due to its high structural heterogeneity, which serves as a benchmark for next-generation modalities, such as Fc-fusion proteins. Unlike standard IgG_1_ therapeutics, cetuximab contains four N-glycosylation sites, two in the Fc region (Asn-299) and two in the Fab region (Asn-88), along with significant charge heterogeneity arising from incomplete C-terminal lysine clipping and sialylation [32].

**Fig. 1:**
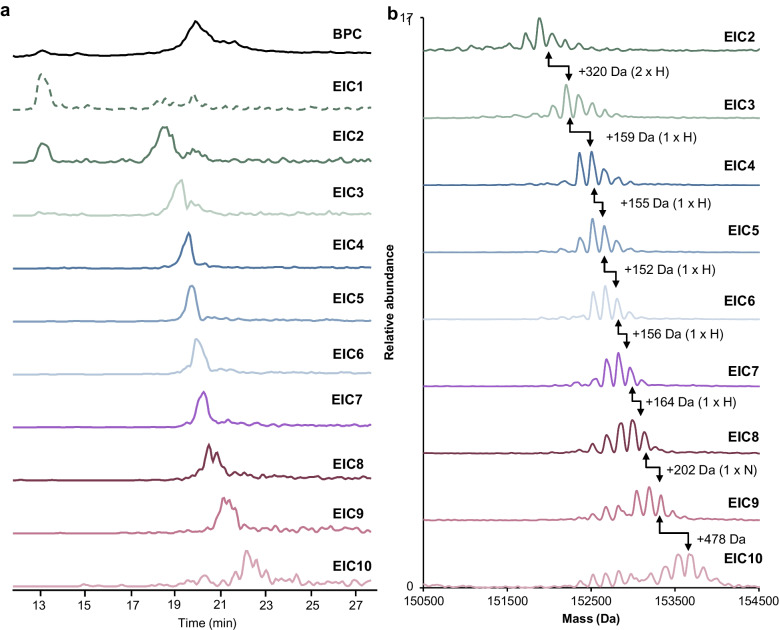
**a** HILIC-MS of direct injection cetuximab. Base peak chromatogram (BPC, *m/z* 3000–6000) and extracted ion chromatograms (EICs) of the glycoform separations are plotted. The glycoforms are illustrated according to ref. [37] (see Table S2 for the glycoform structures). Differences in glycoform combinations among the EICs are highlighted in color. Isomeric structures are possible but not illustrated here. **b** Deconvoluted spectra of the EICs, with the mass difference between the most abundant monosaccharide peak in each spectrum, are annotated. H is hexose, and N is N-acetylglucosamine. Details on the EIC traces are collected in Table S3

We first evaluated the performance of our recently developed low-flow HILIC-MS method [31] (i) by performing direct analysis of intact cetuximab. The results revealed several glycoform distributions (Fig. 1). However, the spectral complexity of the different elution areas was high, precluding the assignment of the observed proteoforms. We, therefore, separated (ii) charge variants (e.g., arising from incomplete C-terminal lysine clipping and sialylation) using IEC-UV, followed by fraction collection. These fractions were then analyzed offline using an RPLC trap column and our (iii) HILIC-MS method. This orthogonal approach deconvolutes the MS spectral complexity observed in the 1D HILIC-MS analysis and enables (iv) comprehensive assignment of glycoforms to specific charge variants.

### Direct Low-flow HILIC-MS of cetuximab

Our previously reported acrylamide-monolithic HILIC-MS method was originally developed for mAbs with only two glycosylation sites [31]. Given the increased polarity and heterogeneity of cetuximab, the method required optimization. The gradient slope was maintained at 0.25%B·min^−1^ (now from 72 to 67%B in 20 min), but the injected mass was increased to 200 ng to detect low-abundance glycoforms separated across a wider retention window.

The HILIC separation was driven by the combined hydrophilicity of the Fab and Fc glycans (Fig. 1A, see Table S2 for the glycosylation nomenclature). Up to nine glycoform elution areas could be resolved within an elution window of 14 to 16 min and 19 to 26 min (Fig. 1 and Table S3). Mass shifts corresponding to single hexose addition (162 Da) were observed in the time window between 19 and 22 min as a broad cluster spanning 152–164 Da (Fig. 1). Similarly, the shift for two hexose units (theoretical 324 Da) was observed at 320 Da. However, due to the high heterogeneity and complexity of cetuximab, many glycoforms observed in extracted ion chromatograms (EICs) could not be identified (Table S4).

Interestingly, two chromatographic elution areas around 13–15 min were observed, with masses of about 148,000 Da. In LC-MS analysis of cetuximab, the main isoform (G0F/G1F as Fc glycans with H7N4F1/H7N4F1 as Fab glycans) appears at about 152,500 Da [34, 36]. The observed mass difference corresponds to two Fab glycans (e.g., H7N4F1/H7N4F1, each ~ 2110 Da). This suggests that our HILIC method detects site-occupancy variants corresponding to Fc-only glycoforms that were previously unobserved. These forms were tentatively assigned to hexose and fucose variants from G0/G0 to G1F/G1F (Fig. S3). Relatively large mass errors (5–16 Da, Table S5) were observed, possibly due to the lower abundance of the species and the possible co-presence of other proteoforms (e.g., charge variants such as deamidation or lysine clipping).

Following the elution of the Fc-only glycans of cetuximab, a distinct chromatographic area containing highly hydrophilic, singly charged species was observed (Fig. S4). The retention and mass profiles suggest these species may represent low-level free glycans initially present in the sample, although it cannot be excluded that they are N-glycans released from the intact protein via on-column hydrolysis in the acidic, high-organic mobile phase.

### Charge variants separation and fractionation using SCX-UV

Cetuximab is a mAb with a relatively basic pI with an isoelectric point in the range of 8.7–8.9 [38]. This biopharmaceutical exhibits significant charge heterogeneity due to PTMs, such as sialylation and C-terminal lysine variability. (ii) To resolve the charge variants, we used SCX chromatography, the standard method for separating charge variants in mAbs [7, 25, 39], and selected MES-NaCl salt gradient buffer based on non-volatile buffer conditions due to its simplicity. Additionally, the method was coupled offline, allowing a desalting step before the HILIC-MS analysis. As the purpose of the experiment was to fractionate the mAb variants, we used a 2.1 mm ID column and operated at reduced flow rates (approximately 0.1 mL·min-1) to reduce the fraction volumes.

**Fig. 2:**
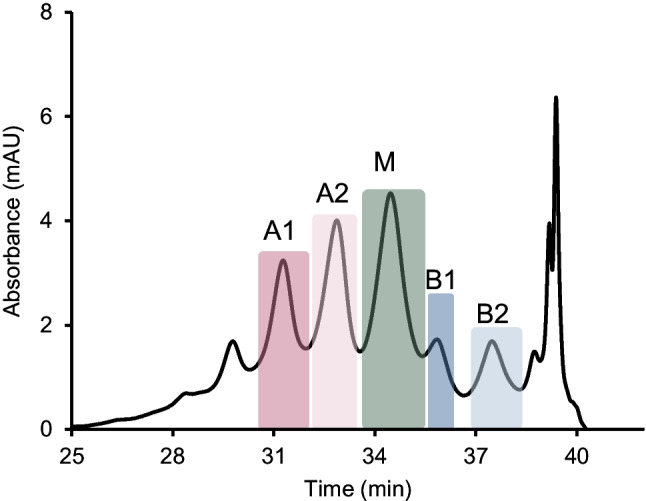
SCX-UV (280 nm detection wavelength) chromatogram of the separation of cetuximab. The fractions taken from these analyses are indicated by the colored squares: acidic variants (A1–A2, pink colored), main isoform (M, green), and basic variants (B1–B2, blue colored). See Tables S1 and S6 for the fraction time windows and the calculated quantities and fraction volumes of the charge variants

We developed a shallow salt gradient (0.6 mM NaCl·min^−1^) SCX method to maximize the resolution between the main isoform peak (M) and its isoforms (Fig. 2). The resulting LC-UV profile revealed extensive heterogeneity: the main isoform accounted for approximately 26.4% of the total peak area, while acidic and basic variants represented ~ 37.0% and ~ 36.6%, respectively. Previous SCX-MS studies have resolved forms based on modifications from both domains: acidic variants are primarily attributed to sialylation of Fab glycans, whereas basic variants are typically attributed to incomplete C-terminal lysine clipping in the Fc domain [34].

From this separation, we isolated five distinct charge variant fractions (acidic: A1, A2; main: M; basic: B1, B2) using an automated fraction collector (Fig. 2 and Table S1), with fraction volumes ranging between 100 and 140 µL (Table S6). Based on the peak areas of the different proteoforms and the injected sample concentration (1 mg·mL^−1^), we estimate that there was significant variation in protein amount between fractions (e.g., fraction estimated concentration of only 1.7 ng·µL^−1^ for B1 vs 9.6 ng·µL^−1^ for M) (Table S6).

It should be noted that smaller column dimensions and lower flow rates increase the influence of extra-column dispersion. This, in combination with the use of a quaternary pump to deliver the SCX mobile phase gradient, may be the reason for the relatively lower separation performance of our IEC method relative to typical separations on 4.6 mm i.d. columns (e.g., in [6, 7]).

### HILIC-MS analysis of cetuximab SCX fractions

Next, we developed an HILIC-MS method to characterize the fractions collected from SCX. The direct analysis of IEC fractions by HILIC-MS is complicated by solvent incompatibility. The IEC fractions contain non-volatile salts in high concentrations (MES, NaCl) and water, whereas HILIC-MS requires high-organic, salt-free conditions for retention (HILIC) and ionization (MS). To bridge this gap without manual desalting steps (which could result in sample loss), we implemented a C4 RPLC trap column to desalt and concentrate our cetuximab SCX fractions before injecting them into a capillary-scale HILIC setup (Fig. S2). Two critical parameters were optimized to ensure efficient transfer: RPLC trap wash volume and the injection volume. RPLC trapping conditions previously used [31], washing the trap after sample loading with 10 µL of mobile phase (0.1% (v/v) TFA in 98:2 water: ACN) proved insufficient for salt removal (Fig. S5). Increasing the trapping-wash volume to 40 µL (with a 4 min wash) effectively eliminated the interfering salts, enabling the successful elution of the protein onto the HILIC-MS dimension.

Given the low concentration of the charge variant fractions (e.g., B1), we increased the injection volume of the fraction to 20 µL, ten times more than the direct analysis of the cetuximab by HILIC-MS. This large-volume injection did not affect chromatographic performance (Fig. S6). Instead, it significantly increased the signal intensity, demonstrating that the trap-based interface preserves separation efficiency while overcoming the sensitivity limitations of fractionated samples.

**Fig. 3:**
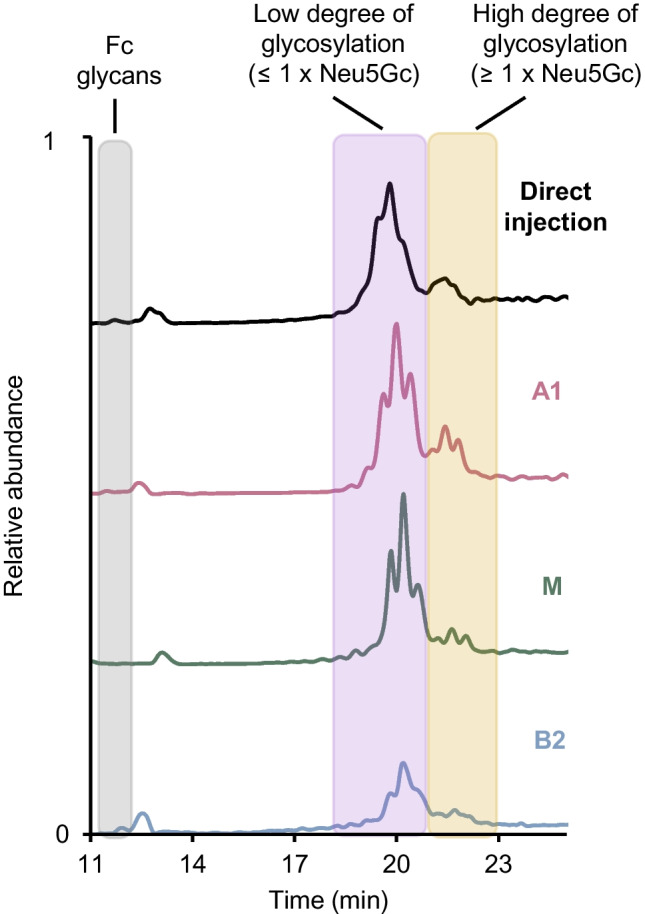
BPC (*m/z* 3000–6000) of the HILIC-MS separation of direct injection cetuximab (black) and the SCX fractions (A1 (pink), M (green), and B2 (blue)). Elution areas of the Fc glycans (gray), low degree of glycosylation (purple), and high degree of glycosylation (yellow) are annotated by squares. The retention times were aligned to the most abundant glycoform in the main fraction (M) (20.21 min). See Table S10 for the real retention times and Figs. S7 and S8 for the glycoform separations

As previously described, HILIC separations are driven by glycoform composition. Interestingly, the glycoform separation profiles were largely comparable across all SCX fractions (Fig. 3). This retention alignment confirms the orthogonality of the two dimensions: the IEC dimension separates based on surface charge (C-terminal lysine variants, deamidation, and sialylation), while the HILIC dimension remains selective for glycans’ hydrophilicity. Figure 3 shows the HILIC-MS profile of A1, M, and B2 fractions.

### Characterization of glycoforms and charge variants by HILIC-MS

The HILIC-MS glycoform separation of the main fraction revealed that up to 15 glycoform peaks could be chromatographically distinguished (Fig. S7 and Tables S7 and S8). For the other fractions, similar separation profiles were obtained (Fig. S8 and Table S9). In contrast, in direct injection of cetuximab, only 9 glycoform peaks were observed (Fig. 1). The separation occurred within a 17–24 min time window and appears to be driven primarily by the number of Fc and Fab glycans present. The mass spectra observed in the SCX fractions were overall simpler than those from direct injection, resulting in more peak features in the base peak chromatogram (Fig. 3).

The deconvolution of the HILIC-MS for the fractionated samples revealed three distinct HILIC elution windows, corresponding to different levels of glycosylation complexity (Fig. 3): Fc-only glycoforms, neutral and singly sialylated glycoforms, and highly sialylated glycoforms. An initial elution area was observed at approximately 12 min (gray region, Fig. 3), which contained species with masses matching the theoretical values for Fc-only glycosylated cetuximab (e.g., G0/G0F and G0F/G0F), indicating the absence of Fab glycans (Fig. S9 and Table S11). The main elution window, between 19 and 21 min (purple region, Fig. 3), was dominated by neutral glycoforms (e.g., G0F/G1F combined with neutral Fab glycans) and singly sialylated species (Table S12). Finally, a third, later-eluting window was observed between 22 and 24 min (yellow region, Fig. 3 and Table S12). This region contained higher-mass species enriched with N-glycolylneuraminic acid (Neu5Gc). Since Neu5Gc introduces both mass (+ 307 Da) and significant polarity, these highly sialylated glycoforms (containing two or more Neu5Gc residues) exhibited the strongest retention on the amide stationary phase (Fig. S10) [40]. This elution behavior confirms that the HILIC selectivity for sialylated species is driven by hydrophilicity, effectively separating them from the earlier-eluting neutral forms.

**Fig. 4:**
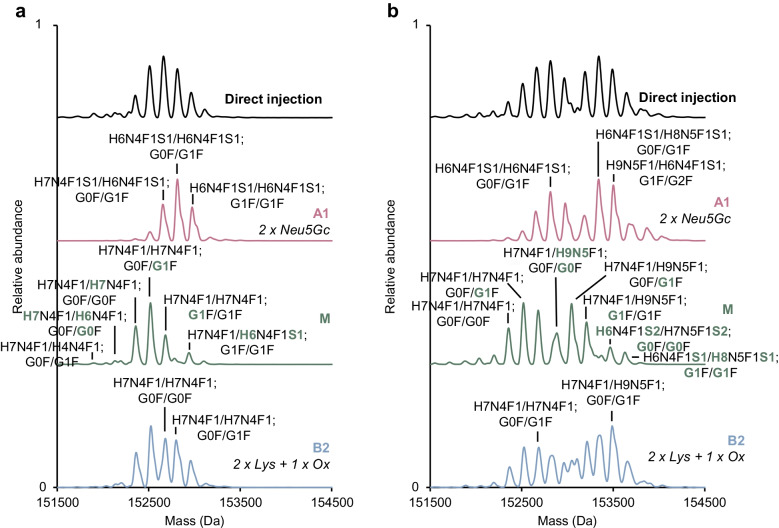
Deconvoluted spectra after HILIC-MS of cetuximab direct injection (black) and SCX fractions (A1 (pink), M (green), and B2 (blue)). The deconvoluted spectrum is calculated based on the average spectrum of **a** elution area purple from Fig. 3 (19.5–21.0 min) and **b** elution area yellow of Fig. 3 (21.5–24.0 min). The glycoforms and charge variants (sialylation (Neu5Gc), C-terminal lysine clipping (Lys), and methionine oxidation (Ox)) are annotated. Isomeric structures are not included. Glycoforms are illustrated according to ref. [37]. See Table S2 for the glycoform structures and Tables S12 and S13 for more structural information

When comparing the deconvoluted masses obtained from different HILIC-MS elution areas, significant spectral differences were observed. The heterogeneous composition of cetuximab proteoforms remains in the SCX fractions, but it is noticeably reduced relative to the direct injection experiment (Fig. 4). To tentatively identify the deconvoluted masses observed, we used the masses reported by Füssl et al*.* [34] and assigned them to specific deconvoluted MS peaks (Table S12). The acidic variants (A1 and A2) contained higher levels of sialylated glycans (ranging from H7N4F1/H6N4F1S1 to H8N5F1S1/H8N5F1S1). This difference was particularly evident in the highly glycosylated elution area observed in HILIC-MS. Cetuximab contains Neu5Gc residues on the Fab glycans, which introduce both a negative charge (causing earlier elution in SCX) and a distinct mass shift. While the additional charge from sialic acids can complicate standard HILIC separations, our orthogonal approach successfully resolved these species. The deconvoluted spectra for fraction A1 (Fig. 4) show a clear enrichment of glycoforms containing one or two Neu5Gc residues, identified by their characteristic mass offsets of 307 Da relative to the main peak (Table S13). This confirms that the acidic nature of these fractions is driven by Fab-localized sialylation. However, overlapping masses were also observed between M and acidic fractions (A1 and A2). Deamidation, another common acidic variant, likely contributes to charge heterogeneity, particularly in acidic regions. However, a +1 Da mass shift (deamidation) is indistinguishable at the intact protein level and would require peptide mapping for confirmation [41]. Additionally, the glycoform modification to a specific glycosylation site could also not be determined at the intact protein level; enzymatic digestion is necessary to achieve this.

In contrast, the basic fractions (B1 and B2) exhibited a glycan profile structurally similar to the main SCX fraction (predominantly neutral G0F/G1F species), but with a systematic mass shift. The most abundant peaks in the B2 fraction were shifted by + 256 Da relative to the main isoform (Fig. 4 and Table S13). This shift corresponds to the presence of two times C-terminal lysine residue (2 × Lys), which retains a positive charge and retards elution on the SCX column. Variants with one C-terminal lysine residue (+ 128 Da) were also detected. Additionally, mass shifts corresponding to methionine oxidation (+ 16 Da) were observed in the basic fractions. While oxidation can be an artifact of sample handling or storage [42], its specific detection here suggests it may alter the surface charge distribution enough to affect IEC retention or co-elute with lysine variants.

When comparing the glycoforms observed with those reported by Füssl et al. [34], it is worth noting that we observed a higher abundance of less glycosylated forms. G0F/G0F and G0F/G1F make up ~ 48% of the total signal in our data, whereas they only represent ~ 15% in Füssl et al*.* data [34]. The dataset of Füssl et al*.* [34] has a much broader spread across the heavily sialylated variants. We believe that this difference in abundance may result from incomplete fractionation during IEC separation (limiting the observed acidic variants) and from differences in ionization efficiency between IEC and HILIC-MS.

## Conclusion

This work expands the applicability of acrylamide-based monolithic stationary phases for the multidimensional separation and structural characterization of the cetuximab (IgG_1_), which carries four N-glycosylation sites (Fab glycans and Fc glycans). The inherent structural heterogeneity of mAbs, arising from a complex mixture of charge variants and glycoforms, necessitates integrated analytical strategies to achieve a comprehensive molecular understanding.

To address this, an orthogonal IEC–(offline)–HILIC–MS workflow was developed, enabling isolation of charge variant fractions (sialylation and C-terminal lysine clipping) by SCX, followed by fractionation and detailed glycoform profiling by HILIC-MS. The implementation of an RP trap column prior to HILIC-MS separation permitted direct sample injection under IEC conditions (*i.e.,* high salt concentration and elevated pH), ensuring method compatibility. Furthermore, the RPLC trap enabled increased sample quantity without compromising chromatographic resolution, thereby facilitating the analysis of low-abundance charge variants using low-flow HILIC-MS.

The results demonstrated that HILIC efficiently resolves both Fc- and Fab-associated glycoforms of cetuximab. Importantly, coupling IEC with HILIC-MS enhanced glycoform resolution beyond that achievable with HILIC-MS alone, providing more detailed insights into charge heterogeneity and glycoform profiling. In addition to the Fc and Fab glycoform combinations, even Fc-only glycoforms were separated from the main isoform. The orthogonality of IEC and HILIC separations affords complementary information on both charge and glycosylation heterogeneity, enabling a more complete structural interpretation of cetuximab. This method can be used to obtain more in-depth information about next-generation protein therapeutics on charge and glycoform heterogeneity.

## Supplementary Information

Below is the link to the electronic supplementary material.

Supplementary file1 Additional information and data on the experimental setup, methodology, and glycoforms and charge variants identification. (PDF 824 KB)

## Data Availability

Raw MS data can be found here: 10.5281/zenodo.18401232.
