# Supplementary material for: Resolving charge and glycosylation variants of cetuximab combining offline ion-exchange and hydrophilic interaction chromatography–HRMS
Source: Anal Bioanal Chem. 2026 Apr 27;418(13):4015–23. doi: 10.1007/s00216-026-06519-w (PMC13264599; doi:10.1007/s00216-026-06519-w)
Supplement: Supplementary file 1 — Supplementary file1 Additional information and data on the experimental setup, methodology, and glycoforms and charge variants identification. (PDF 824 KB) [file 216_2026_6519_MOESM1_ESM.pdf]

ELECTRONIC SUPPLEMENTARY INFORMATION

Resolving Charge and Glycosylation Variants of Cetuximab Combining offline Ion-Exchange and Hydrophilic Interaction Chromatography – HRMS

Annika A.M. van der Zon[1,2] and Andrea F.G. Gargano[1,2,*]

[1] University of Amsterdam, van 't Hoff Institute for Molecular Sciences, Analytical Chemistry Group, Science Park 904, 1098 XH Amsterdam, The Netherlands

[2] Center of Analytical Sciences Amsterdam, Science Park 904, 1098 XH Amsterdam, The Netherlands

[*] Correspondance: a.gargano@uva.nl

Contents

S-1 IEC-HILIC-MS setup ____ 3

S-2 Nomenclature Fc and Fab glycoforms ____ 5

S-3 Intact glycoforms separation of direct-injection cetuximab ____ 7

S-4 Separation charge variants of cetuximab ____ 9

S-5 HILIC-MS analysis of cetuximab SCX fractions ____ 10

S-6 Characterization of glycoforms and charge variants by HILIC-MS ____ 11

References____ 20

S-1 IEC-HILIC-MS setup

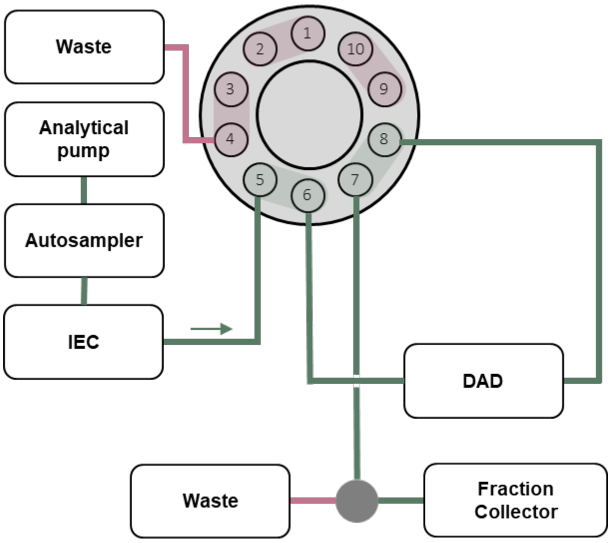

Fig. S1: Schematic representation of the IEC separation with online fractionation

Table S1: Time windows of the fraction collection of cetuximab

Fraction	Time window (min)
A1	30.76 – 31.99
A2	32.28 – 33.47
M	33.83 – 35.21
B1	35.46 – 36.48
B2	37.02 – 38.11

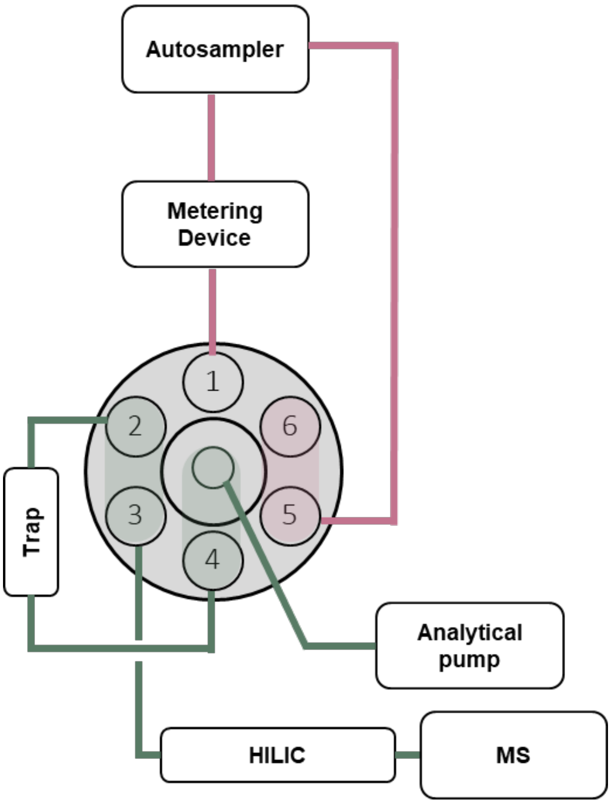

Fig. S2: Schematic representation of the trap-to-elute strategy for HILIC-MS

S-2 Nomenclature Fc and Fab glycoforms

Table S2: Nomenclature and symbolic representations of glycoforms in the Fc and Fab region. The glycoforms are illustrated according to [1].

Structure	Name Fc region	Name Fab region
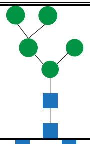	M5	-
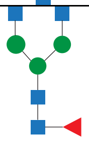	G0F	H3N4F1
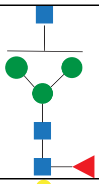	G0F-N	-
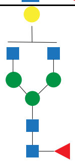	G1F	H4N4F1
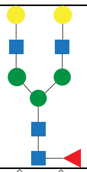	G2F	H5N4F1
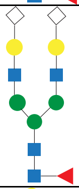	-	H5N4F1S2
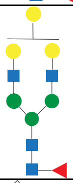	-	H6N4F1
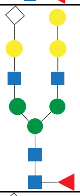	-	H6N4F1S1
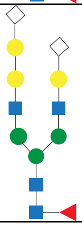	-	H6N4F1S2

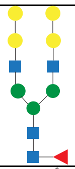	-	H7N4F1
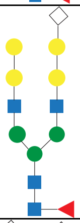	-	H7N5F1S1
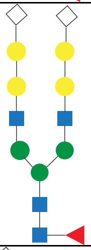	-	H7N5F1S2
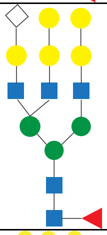	-	H8N5F1S1
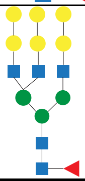	-	H9N5F1

*Mannose (H) is highlighted as a yellow circle, galactose (H) as a green circle, fucose (F) as a red square, N-glycolylneuraminic acid (S) as a white diamond, and N-Acetylglucosamine (N) as a blue square.

S-3 Intact glycoforms separation of direct-injection cetuximab

Table S3: The *m/z* values for generating the glycoforms EICs of the cetuximab standard. A Gaussian smoothing parameter of 7 was applied with a mass tolerance of 0.50 amu.

EIC	Time window (min)	m/z
1	10.09-10.46	3795.9035,3895.8249,4001.1579,4112.2734
2	15.13-15.56	3895.8155,3997.6153,4105.5536,4219.5636
3	15.70-16.13	3903.2077,4006.0861,4114.1511,4229.0102
4	16.13-16.42	3907.3855,4010.1533,4118.4930,4232.7816
5	16.39-16.61	3911.2481,4014.3039,4122.6316,4237.3334
6	16.61-16.88	3915.5930,4018.6158,4217.4017,4241.8562
7	16.80-17.09	3919.4577,4022.8315,4131.4285,4246.0481
8	17.23-17.63	3924.0615,4027.5442,4135.6375,4251.7340
9	17.95-18.19	3932.4693,4032.2592,4141.1624,4256.5829
10	18.83-19.23	4266.7510,4392.5593,4521.5879,4662.7255

Table S4: Identification of Fab and Fc glycoforms of cetuximab standard with HILIC-MS. The theoretical masses are based on [2] . Isomeric structures are not included. The EIC traces are generated according to Table S3.

EIC	Fab glycoforms	Fc glycoforms	Theoretical mass (Da)	Measured mass (Da)	Mass error (Da)
1	-	G0/G0F	148,018.92	148,179.6	-15.5
2	-	-	-	-	-
3	-	-	-	-	-
4	H7N4F1/H7N4F1	G0F/G0F	152,352.2	152,360.6	-8.3
5	H7N4F1/H4N4F1	G0F/G1F	152,514.4	152,515.8	-1.4
6	H7N4F1/H7N4F1	G1F/G1F	152,676.5	152,668.0	8.5
7	H7N4F1/H6N4F1S1	G1F/G1F	152,821.6	152,824.2	-2.6
8	H7N4F1/H9N5F1	G0F/G0F	153,007.9	152,989.1	-5.3
9	H9N5F1/H6N4F1S1	G0F/G1F	153,186.9	153,191.6	-4.7
10	-	-	-	-	-

*Due to the four glycosylation sites of cetuximab, the nomenclature for the N-glycoforms of the Fc and Fab regions is represented in Table S2. The N-glycoforms in the Fab region are assigned as: H3N4F1, H4N4F1, H5N4F1, H7N4F1, and H9N4F1. H is Hexose, N is N-Acetylglucosamine, F is fucose, and S is N-glycolylneuraminic acid.

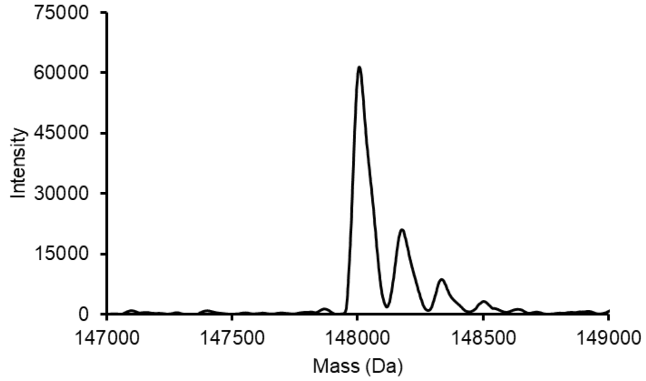

Fig. S3: Deconvoluted spectrum of the elution of the tentatively assigned Fc-only glycoforms of cetuximab sample. The deconvoluted spectrum is created from a time window of 13.8-14.9 min. See Table S5 for the identification of the Fc glycans.

Table S5: Identification of Fc-only glycoforms of cetuximab standard with HILIC-MS. The theoretical masses are based on [2]. Isomeric structures are not included.

Fc glycoform	Theoretical mass (Da)	Measured mass (Da)	Mass error (Da)
G0/G0	147,872.8	147,867.9	4.9
G0/G0F	148,018.9	148,006.6	12.3
G0F/G0F	148,165.1	148,175.9	-10.8
G0F/G1F	148,327.2	148,334.5	-7.3
G1F/G1F	148,489.3	148,500.7	-11.4

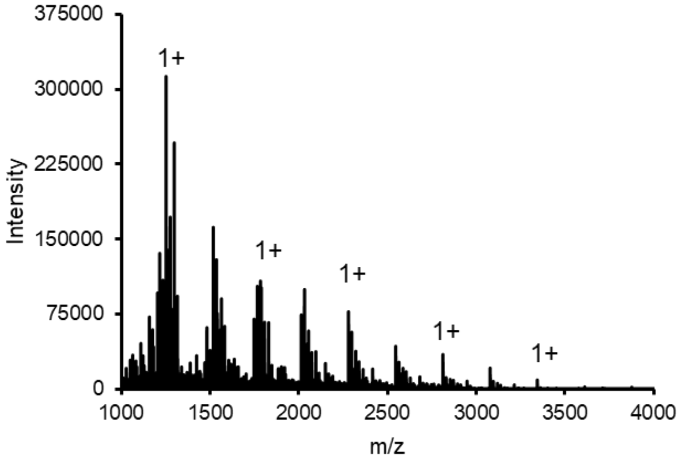

Fig. S4: Raw *m/z* spectrum of the main fraction (M) (time window 13.0-13.2 min). Single-charged peaks are present, which might correspond to single Fab glycans.

S-4 Separation charge variants of cetuximab

Table S6: The estimated quantities and fraction volumes of the charge variants of cetuximab.

Charge variant	Pseudo-quantification (ng)	Fraction volume (µL)
A1	594.28	123.01
A2	972.12	112.02
M	1318.78	138.01
B1	176.08	102.01
B2	392.52	109.01

S-5 HILIC-MS analysis of cetuximab SCX fractions

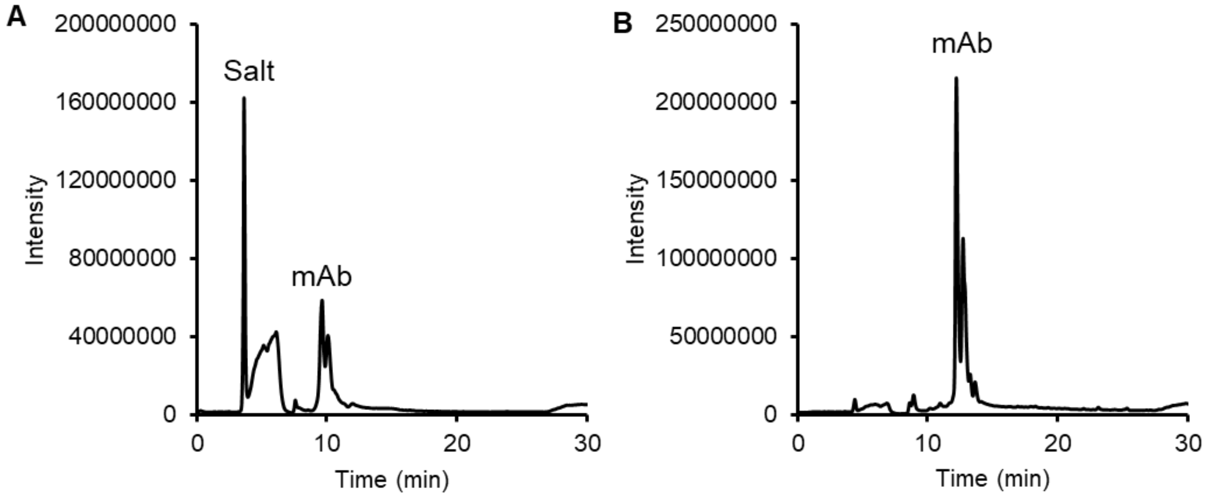

Fig. S5: Separation of mAb-1 with a trapping volume of **(A)** 10 µL and **(B)** 40 µL. There is an intense salt peak in (A), indicating that the trapping volume for desalting the sample was too low compared to (B).

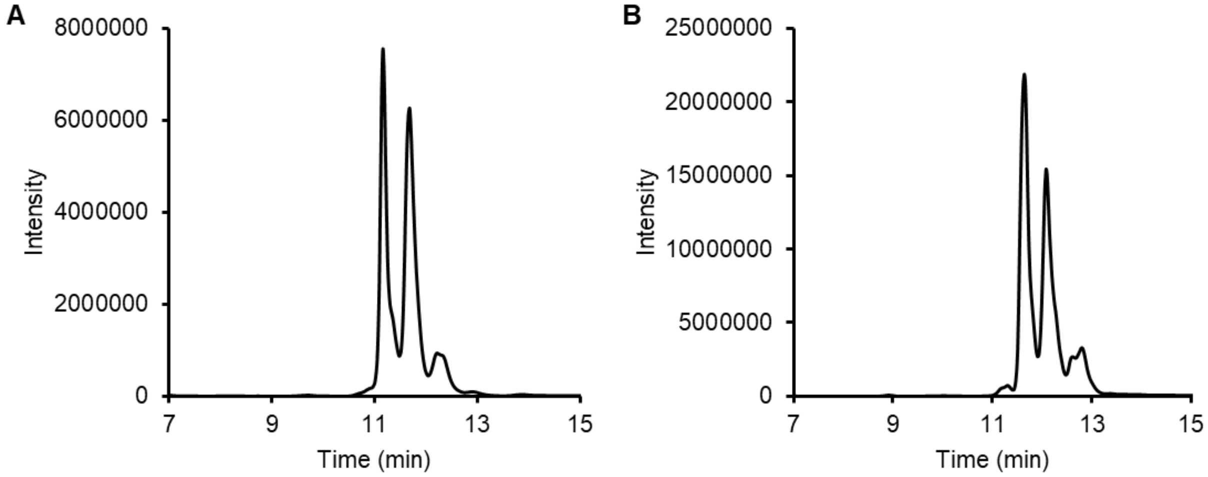

Fig. S6: Separation of mAb-1 with different sample volumes: **(A)** 100 ng (100 ng·µL^{-1}, 1 µL) and **(B)** 105 ng (15 ng·µL^{-1}, 7 µL).

S-6 Characterization of glycoforms and charge variants by HILIC-MS

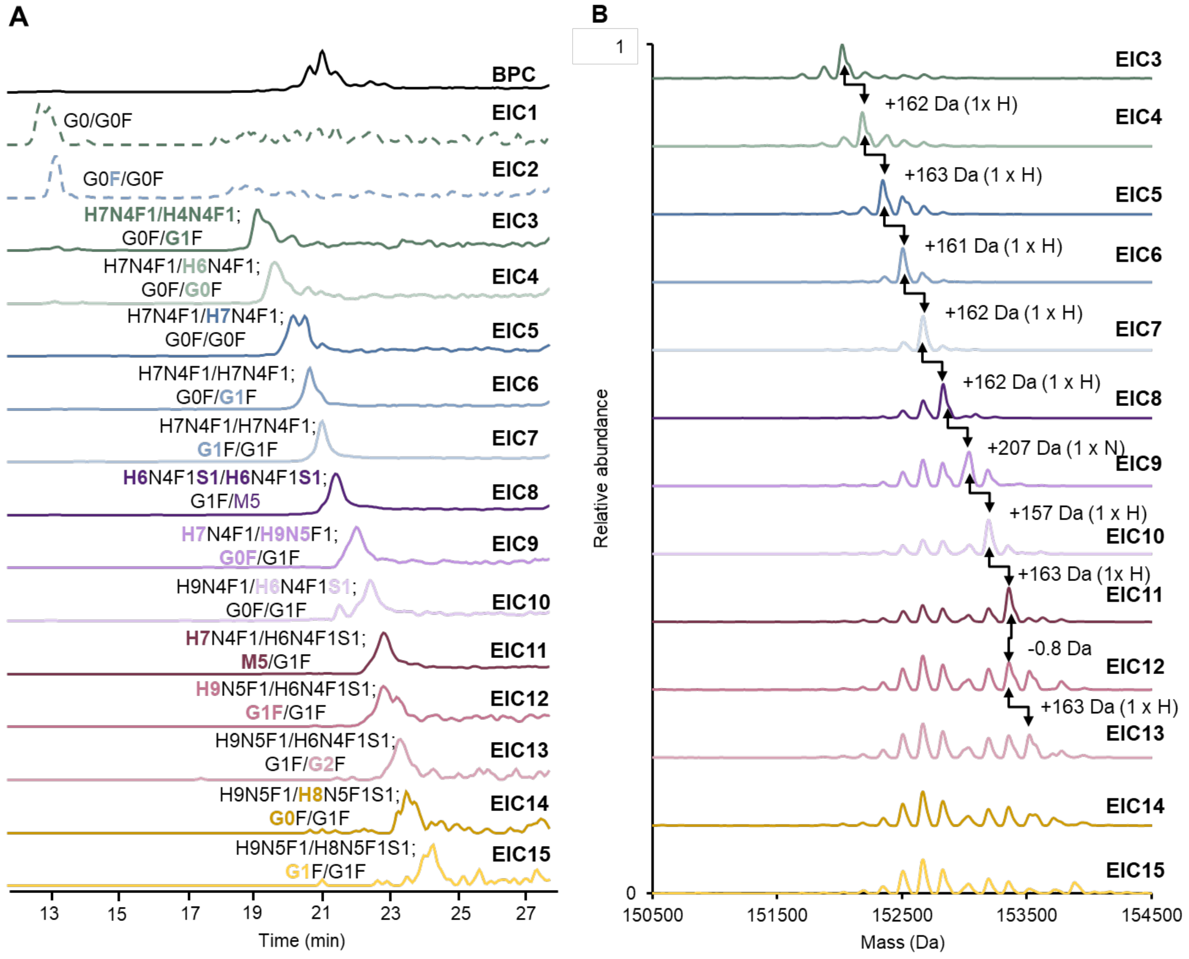

Fig. S7: **(A)** HILIC-MS of cetuximab main isoform (M). BPC (*m/z* 3,000-6,000) and EICs of the glycoform separations are plotted. The glycoforms are illustrated according to ref. [1] (see Table S2 for the glycoform structures). Differences in glycoform combination between the EICs are highlighted by color. Isomeric structures are possible but not illustrated here. **(B)** Deconvoluted spectra of the EICs, with the mass difference between the most abundant monosaccharide peak in each spectrum, are annotated. H is hexose, and N is N-acetylglucosamine. Details on the EIC traces are represented in Table S7.

Table S7: The *m/z* values for generating the glycoforms EICs of the cetuximab main isoform fraction. A Gaussian smoothing parameter of 7 was applied with a mass tolerance of 0.50 amu.

EIC	Time window (min)	m/z
1	12.80-13.09	3610.9452,3702.1414,3796.6968,3896.7491
2	13.22-13.45	3528.6820,3614.8183,3705.0045,3800.0380
3	19.25-19.48	3708.8690,3801.4439,3898.9855,4001.5099
4	19.72-20.03	3712.6995,3805.4801,3903.0609,4005.7446
5	20.37-20.76	3716.6386,3809.5767,3907.1704,4009.9381
6	20.79-20.99	3720.5056,3813.5137,3911.2474,4014.2171
7	21.12-21.33	3724.5911,3817.6822,3915.5319,4018.5655
8	21.57-21.80	3728.5114,3821.7387,3919.6933,4022.8377
9	22.14-22.40	3733.6833,3826.8413,3925.0402,4028.2124
10	22.53-22.84	3737.3885,3830.7721,3928.9571,4023.3059
11	22.94-23.26	3741.3188,3834.8200,3933.2146,4036.6604
12	23.10-23.46	4149.9300,4265.4040,4382.6645,4511.3579
13	23.41-23.65	4206.2913,4387.7318,4515.7932,4654.4066
14	23.67-24.01	4266.7510,4392.5593,4521.5879,4662.7255
15	24.35-24.61	4274.3676,4396.9451,4526.8250,4662.6610

Table S8: Identification of Fab and Fc glycoforms of main isoform fraction with HILIC-MS. The theoretical masses are based on [2]. Isomeric structures are not included. The EIC traces are generated according to Table S7.

EIC	Fab glycoforms	Fc glycoforms	Theoretical mass (Da)	Measured mass (Da)	Mass error (Da)
1	-	G0/G0F	148,018.9	148,001.4	-17.5
2	-	G0F/G0F	148,165.1	148,166.6	-1.7
3	H7N4F1/H4N4F1	G0F/G1F	152,020.4	152,027.9	7.5
4	H7N4F1/H6N4F1	G0F/G0F	152,190.1	152,182.2	7.9
5	H7N4F1/H7N4F1	G0F/G0F	152,352.2	152,345.1	7.0
6	H7N4F1/H7N4F1	G0F/G1F	152,514.4	152,506.6	7.8
7	H7N4F1/H7N4F1	G1F/G1F	152,676.5	152,668.6	7.9
8	H6N4F1S1/H6N4F1S1	M5/G1F	152,832.7	152,830.1	2.6
9	H7N4F1/H9N5F1	G0F/G1F	153,036.9	153,041.8	4.9
10	H7N4F1/H9N5F1	G1F/G1F	153,204.0	153,193.9	10.1
11	H6N4F1S1/H9N5F1	G1F/G1F	153,349.1	153,356.6	-7.5
12	H6N4F1S1/H7N5F1	M5/G1F	153,360.1	153,356.6	3.6
13	H9N5F1/H6N4F1S1	G1F/G2F	153,511.2	153,520.7	-9.5
14	H9N5F1/H8N5F1S1	G0F/G1F	153,714.4	153,715.1	-0.7
15	H9N5F1/H8N5F1S1	G1F/G1F	153,876.5	153,883.0	-6.5

*Due to the four glycosylation sites of cetuximab, the nomenclature for the N-glycoforms of the Fc and Fab regions are represented in Table S2. The N-glycoforms in the Fab region are assigned as: H3N4F1, H4N4F1, H5N4F1, H7N4F1, and H9N4F1. H is Hexose, N is N-Acetylglucosamine, F is fucose, and S is N-glycolylneuraminic acid.

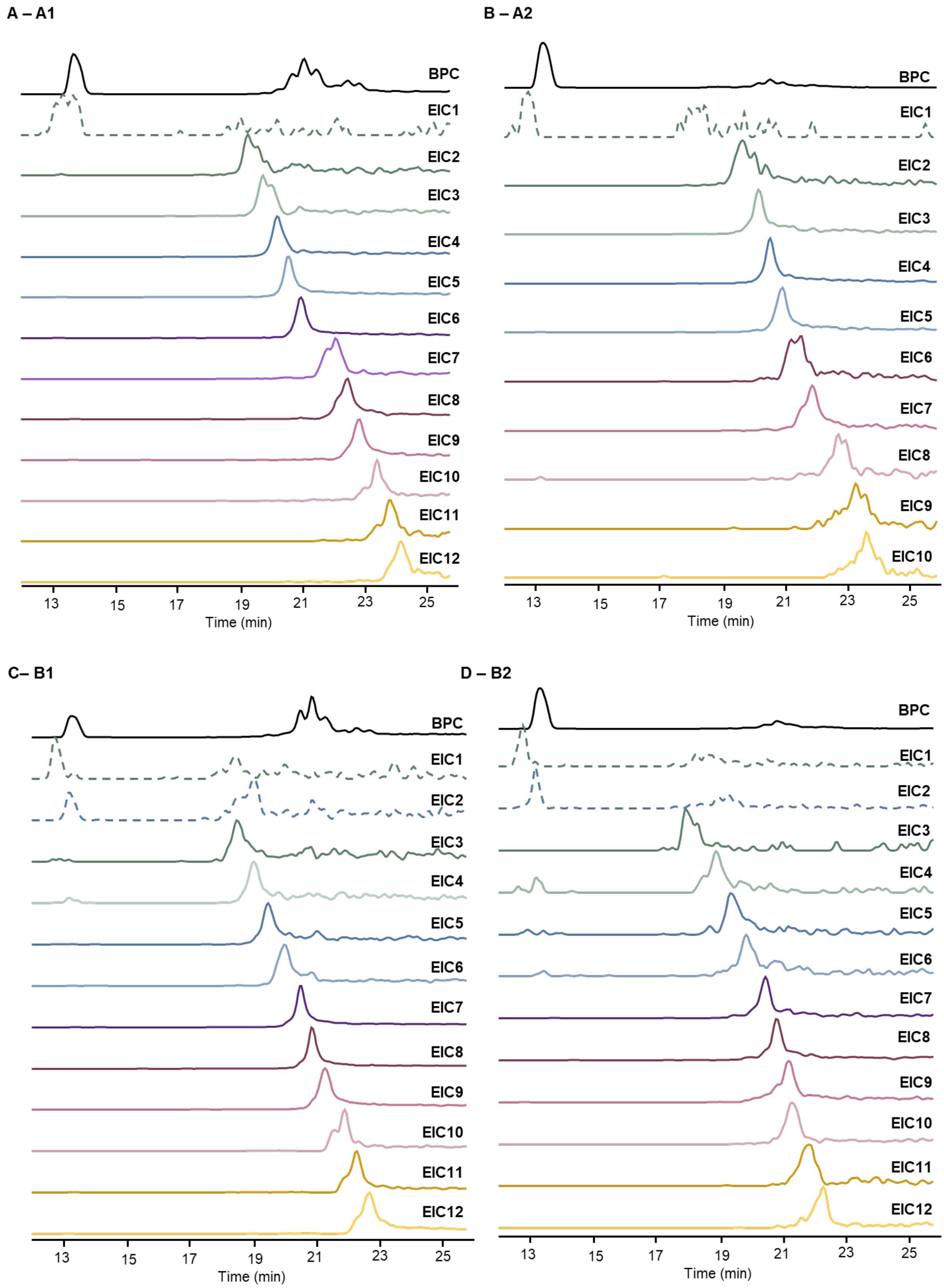

Fig. S8: BPC and EICs of HILIC glycoforms separation of the SCX fractions **(A)** A1, **(B)** A2, **(C)** B1, and **(D)** B2. The traces of the EICs are plotted in Table S9.

Table S9: The *m/z* values for generating the glycoforms EICs of cetuximab charge variant fractions. A Gaussian smoothing parameter of 7 was applied with a mass tolerance of 0.50 amu.

EIC	A2	A3	B1	B2
1	3619.4782,<br>3705.2603,<br>3799.9864,<br>3900.3135	3529.0039,<br>3614.9280,<br>3705.2999,<br>3799.8559	3524.7043,<br>3610.7676,<br>3700.7691,<br>3795.9179	3611.4109,<br>3701.2131,<br>3796.0565,<br>3896.8481
2	3627.8208,<br>3716.2237,<br>3809.1743,<br>3906.8077	3628.2578,<br>3716.7460,<br>3907.2411,<br>4010.0372	3529.4288,<br>3614.8149,<br>3705.1044,<br>3799.7021	3614.5307,<br>3704.8166,<br>3800.0428,<br>3900.9059
3	3631.6717,<br>3720.2900,<br>3813.2334,<br>3910.9594	3632.0312,<br>3720.6704,<br>3813.6367,<br>3911.4024	3613.1479,<br>3701.1953,<br>3793.7322,<br>3890.9928	3697.3694,<br>3794.7868,<br>3886.8484,<br>3989.3381
4	3635.5274,<br>3724.1771,<br>3817.2834,<br>3915.1004	3635.9223,<br>3724.5811,<br>3817.7044,<br>3915.5371	3617.0116,<br>3705.2308,<br>3797.8479,<br>3895.1477	3895.2257,<br>3705.2071,<br>3793.8756,<br>3997.9762
5	3639.4193,<br>3728.1282,<br>3821.3133,<br>3919.2622	3639.8086,<br>3728.5089,<br>3821.7310,<br>3919.6604	3620.7875,<br>3709.0991,<br>3801.8499,<br>3899.2578	3801.8703,<br>3899.3560,<br>4001.9513,<br>3709.3883
6	3643.2022,<br>3732.0741,<br>3825.3275,<br>3923.4328	3644.5678,<br>3733.5168,<br>3826.8737,<br>3924.9183	3624.6850,<br>3713.0479,<br>3805.9295,<br>3903.4389	3713.0562,<br>3805.8432,<br>3903.4852<br>,4006.2643
7	3648.1607,<br>3737.0187,<br>3830.4467,<br>3928.7683	3648.3553,<br>3737.3466,<br>3830.6847,<br>3929.0175	3628.5377,<br>3716.9030,<br>3809.8253,<br>3907.6027	3717.0785,<br>3810.1048,<br>3907.6644,<br>4010.4281
8	3651.7564,<br>3740.9286,<br>3834.4591,<br>3932.7554	3652.3696,<br>3741.3286,<br>3834.9976,<br>3933.2096	3632.4138,<br>3720.9782,<br>3814.0127,<br>3911.7948	3721.0532,<br>3814.0209,<br>3911.8394,<br>4014.7527
9	3655.7390,<br>3744.9510,<br>3838.5491,<br>3936.9321	3656.5422,<br>3746.3156,<br>3840.1671,<br>3938.9213	3641.1987,<br>3729.9882,<br>3823.0699,<br>3921.0536	3724.9970,<br>3818.0778,<br>3915.8367,<br>4019.0342
10	3749.9760,<br>3843.6904,<br>3942.2959,<br>4046.0767	3660.9626,<br>3751.5175,<br>3848.0653,<br>3942.3469	3644.8519,<br>3733.7406,<br>3827.1111,<br>3925.2510	3733.0793,<br>3825.0129,<br>3923.0304,<br>4026.3146
11	3753.8229,<br>3847.5895,<br>3946.3819,<br>4049.9405	-	3648.9295,<br>3737.8142,<br>3831.2353,<br>3929.4317	3737.1065,<br>3830.3850,<br>3928.3129,<br>4031.5748
12	3757.7467,<br>3851.6364,<br>3950.3868,<br>4054.4041	-	-	3740.8189,<br>3834.1060,<br>3932.3646,<br>4035.6372

Table S10: Retention times of the most abundant glycoform peak of cetuximab from the charge variant fractions.

Fraction	Time (min)
A1	20.76
A2	20.57
M	20.21
B1	21.00
B2	20.97

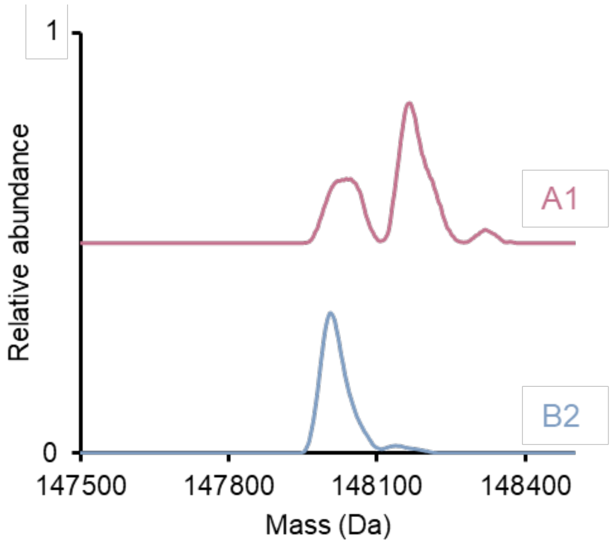

Fig. S9: Deconvoluted spectrum of Fc-only glycoforms of SCX fraction A1 (pink) and B2 (blue). The deconvoluted spectrum is created from a time window of 12.1-12.9 min. See Table S11 for the identification of the Fc glycans.

Table S11: Identification of Fc-only glycoforms of A1 with HILIC-MS. The theoretical masses are based on [2]. Isomeric structures are not included.

Fraction	Fc glycoform	Theoretical mass (Da)	Measured mass (Da)	Mass error (Da)
A1	G0/G0F	148,018.9	148,021.5	-2.6
	G0F/G0F	148,165.1	148,165.8	-0.7
	G0F/G1F	148,327.2	148,320.1	-7.1
B1	G0/G0F	148,018.9	148,005.7	-13.2
	G0F/G0F	148,165.1	148,165.9	-0.8

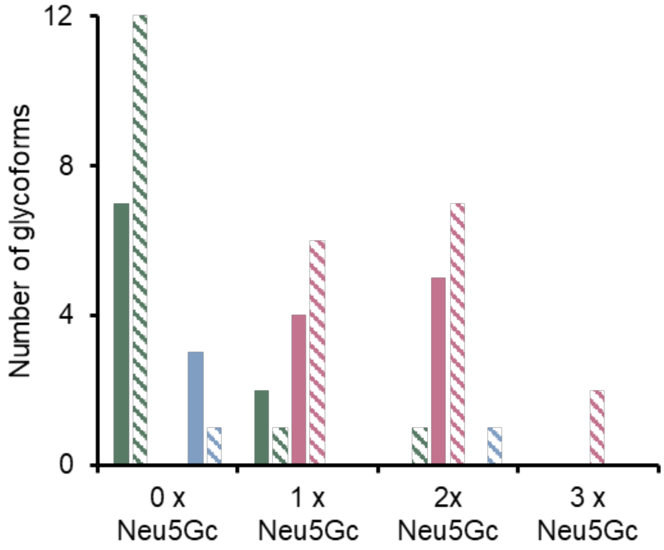

Fig. S10: The number of sialylated glycoforms of the SCX fractions (A1 (pink), M (green), and B2 (blue)) characterized at two time windows: 19.5-21.5 min (non-striped colored, lower degree glycosylation) and 21.5-23.5 min (striped colored, higher degree glycosylation).

Table S12: Identification of glycoforms of the cetuximab fractions after IEC-HILIC-MS. The deconvoluted spectra are generated from a time window of 17-24 min. The theoretical masses are based on [2]. The maximum mass error was set to 10 Da. Some masses with abundances above 10% were not identified. "N.A." means "not assessed."

Fraction	Fab glycoforms*	Fc glycoforms*	Theoretical mass (Da)	Measured mass (Da)	Mass error (Da)	Abundance (%)	Time (min)
A1	H7N4F1/H6N4F1	G0F/G0F	152,190.1	152,191.6	-1.5	2.4	18.5
	H7N4F1/H7N4F1	G0F/G0F	152,352.2	152,350.7	1.5	8.6	19.5
	H7N4F1/H7N4F1	G0F/G1F	152,514.4	152,506.8	7.6	22.9	20.0
	H7N4F1/H6N4F1S1	G0F/G1F	152,659.5	152,653.8	5.7	64.2	20.5
	H6N4F1S1/H6N4F1S1	G0F/G1F	152,803.3	152,814.0	-10.7	100.0	20.9
	H6N4F1S1/H6N4F1S1	G1F/G1F	152,966.7	152,975.5	-8.8	67.5	21.0
	H7N4F1/H6N4F1S1	G1F/G2F	152,983.8	152,975.5	8.3	67.5	21.0
	H8N5F1S1/H6N4F1S1	G0F/G0F	153,169.9	153,179.6	-9.7	34.9	21.5
	H6N4F1S1/H8N5F1S1	G0F/G1F	153,332.1	153,338.9	-6.8	42.1	22.0
	H9N5F1/H6N4F1S1	G1F/G2F	153,511.2	153,501.4	9.8	30.1	22.5
	N.A.	N.A.	-	153,674.9	-	9.1	23.0
	H9N5F1/H8N5F1S1	G1F/G1F	153,876.6	153,870.2	6.4	7.6	23.5
	H8N5F1S1/H8N5F1S1	G1F/G1F	154,021.7	154,030.6	-8.9	5.4	23.5
A2	H7N4F1/H4N4F1	G0F/G1F	152,027.9	152,031.8	-3.9	3.5	18.0
	H7N4F1/H6N4F1	G0F/G0F	152,190.1	152,190.6	-0.5	6.9	19.0
	H7N4F1/H7N4F1	G0F/G0F	152,352.2	152,358.3	-6.1	19.8	19.1
	H7N4F1/H7N4F1	G0F/G1F	152,514.4	152,511.4	3.0	66.7	19.5
	H7N4F1/H7N4F1	G1F/G1F	152,676.5	152,670.7	5.8	100.0	17.8
	H6N4F1S1/H6N4F1S1	G1F/M5	152,832.7	152,832.8	-0.1	69.4	20.8
	H9N5F1/H6N4F1S1	G0F/G0F	153,024.8	153,031.3	-6.5	41.2	21.0
	H7N4F1/H9N5F1	G0F/G1F	153,041.8	153,031.3	10.5	41.2	21.5
	H9N5F1/H6N4F1S1	G0F/G1F	153,186.9	153,195.2	-8.3	42.8	21.5
	H7N4F1/H9N5F1	G1F/G1F	153,204.0	153,195.2	8.8	42.8	21.5
	H6N4F1S1/H8N5F1S1	G1F/M5	153,360.2	153,360.6	-0.4	29.5	22.5
	N.A.	N.A.	-	153,529.0	-	11.5	22.5
	H9N5F1/H8N5F1S1	G1F/G1F	153,876.6	153,883.2	-6.6	4.5	23.5
M	H4N4F1/H4N4F1	G0F/G0F	151,379.4	151,387.9	-8.6	1.6	17.1
	H7N4F1/H3N4F1	G0F/G0F	151,703.6	151,712.5	-8.9	4.5	17.6
	H7N4F1/H4N4F1	G0F/G1F	152,027.9	152,035.4	-7.5	11.1	18.6
	H7N4F1/H7N4F1	G0F/M5	152,124.0	152,129.6	-5.6	4.1	18.0
	H7N4F1/H6N4F1	G0F/G0F	152,190.1	152,196.3	-6.2	16.6	19.0
	H7N4F1/H7N4F1	G0F/G0F	152,352.2	152,358.0	-5.8	67.4	19.5
	H7N4F1/H7N4F1	G0F/G1F	152,514.4	152,519.5	-5.1	100.0	20.0

	H7N4F1/H7N4F1	G1F/G1F	152,676.5	152,681.9	-5.4	61.4	20.5
	H6N4F1S2/H6N4F1S1	G0F/G0F	152,787.6	152,782.6	5.0	6.5	20.0
	H7N4F1/H6N4F1S1	G0F/G1F	152,787.6	152,782.6	5.0	6.5	20.0
	H7N4F1/H9N5F1	G0F/G0F	152,879.7	152,873.8	5.9	15.0	21.2
	H6N4F1S1/H6N4F1S1	G0F/G1F	152,932.8	152,938.0	-5.2	17.1	20.5
	H7N4F1/H9N5F1	G0F/G1F	153,041.8	153,045.0	-3.2	20.4	21.2
	H5N4F1S2/H6N4F1S2	G0F/G1F	153,094.8	153,095.9	-1.1	15.5	21.0
	H6N4F1S1/H6N4F1S1	G1F/G1F	153,094.9	153,095.9	-1.0	15.5	21.0
	H7N4F1/H9N5F1	G1F/G1F	153,204.0	153,209.1	-5.1	15.7	22.1
	H6N4F1S2/H7N5F1S2	G0F/G0F	153,460.1	153,465.7	-5.6	5.4	21.5
	H7N5F1S2/H6N4F1S2	G0F/G1F	153,622.3	153,626.9	-4.6	4.8	22.1
	H6N4F1S1/H8N5F1S1	G1F/G1F	153,622.4	153,626.9	-4.5	4.8	22.1
	H6N4F1S2/H7N5F1S2	G1F/G1F	153,784.4	153,789.5	-5.1	1.7	22.8
B1	H7N4F1/H4N4F1	G0F/G1F	152,156.1	152,166.8	-10.7	15.0	18.5
	H4N4F1/H6N4F1S2	G0F/G1F	152,318.2	152,327.1	-8.9	67.0	20.0
	H7N4F1/H6N4F1	G0F/G0F	152,479.9	152,489.1	-9.2	100.0	20.0
	H7N4F1/H6N4F1S1	G0F/G1F	152,659.5	152,652.6	6.9	59.7	21.5
	H7N4F1/H6N4F1S1	G0F/G0F	152,753.7	152,747.2	6.5	6.5	21.5
	N.A.	N.A.	-	152,846.6	-	17.1	21.4
	H6N4F1S1/H6N4F1S1	G0F/G0F	152,898.8	152,905.6	-6.8	17.1	21.0
	H7N4F1/H6N4F1S1	G0F/G1F	152,915.8	152,905.6	10.2	17.1	21.0
	H7N4F1/H9N5F1	G0F/G0F	153,007.8	153,013.7	-5.9	22.4	21.2
	H6N4F1S1/H6N4F1S1	G0F/G1F	153,060.9	153,065.4	-4.5	16.6	22.0
	N.A.	N.A.	-	153,178.9	-	16.7	22.7
	H6N4F1S1/H9N5F1	G1F/G1F	153,605.4	153,597.3	8.1	4.8	23.0
B2	H7N4F1/H6N4F1S1	G0F/G1F	152,659.5	152,652.1	7.4	87.9	21.2
	H7N4F1/H7N4F1	G0F/G1F	152,770.7	152,772.0	-1.3	65.3	21.0
	H7N4F1/H7N4F1	G1F/G1F	152,932.8	152,928.4	4.4	58.2	21.4
	H7N4F1/H9N5F1	G1F/G0F	153,095.0	153,091.2	3.8	58.2	22.1
	H6N4F1S1/H6N4F1S1	G1F/G1F	153,094.9	153,091.2	3.6	23.9	22.1
	H6N4F1S1/H6N4F1S1F	G1F/G2F	153,128.9	153,131.4	-2.5	25.2	21.7
	H7N4F1/H9N5F1	G0F/G1F	153,298.2	153,302.1	-3.9	24.9	22.1

*Due to the four glycosylation sites of cetuximab, the nomenclature for the N-glycoforms of the Fc and Fab regions is represented in Table S2. For the Fab glycans: H is Hexose, N is N-Acetylglucosamine, F is fucose, and S is N-glycolylneuraminic acid.

Table S13: Identification of the charge variants of the cetuximab fractions after IEC-HILIC-MS. The deconvoluted spectra are created based on a time window of 17-24 min. The theoretical masses are based on [2]. The maximum mass error was set to 10 Da.

Fraction	Fab glycoforms*	Fc glycoforms	Theoretical mass (Da)	Measured mass (Da)	Mass error	Charge variants**
A1	H6N4F1S1/H8N5F1S1	G0F/G1F	152,803.3	152,814.0	-8.7	2 x Neu5Gc
A2	H9N5F1/H6N4F1S1	G0F/G1F	153,186.9	153,195.2	-8.3	1 x Neu5Gc
M	H7N4F1/H7N4F1	G0F/G1F	152,514.4	152,519.5	-5.1	-
B1	H7N4F1/H7N4F1	G0F/G1F	152,658.4	152,652.6	5.8	1 x Lys + 1 x Ox
B2	H7N4F1/H7N4F1	G0F/G1F	152,786.7	152,779.0	7.4	2 x Lys + 1 x Ox

*Due to the four glycosylation sites of cetuximab, the nomenclature for the N-glycoforms of the Fc and Fab regions is represented in Table S2. For the Fab glycans: H is Hexose, N is N-Acetylglucosamine, F is fucose, and S is N-glycolylneuraminic acid. ** Neu5Gc is N-glycolylneuraminic acid sialylation, Lys is C-terminal lysine clipping, and Ox is methionine oxidation.

References

[1] Neelamegham S, Aoki-Kinoshita K, Bolton E, Frank M, Lisacek F, Lütteke T, et al. Updates to the Symbol Nomenclature for Glycans guidelines. Glycobiology 2019;29:620–4. https://doi.org/10.1093/glycob/cwz045.

[2] Füssl F, Trappe A, Carillo S, Jakes C, Bones J. Comparative Elucidation of Cetuximab Heterogeneity on the Intact Protein Level by Cation Exchange Chromatography and Capillary Electrophoresis Coupled to Mass Spectrometry. Anal Chem 2020;92:5431–8. https://doi.org/10.1021/acs.analchem.0c00185.
